# Female-Type Presentation of Male Breast Cancer in Mammography and Its Clinical Implications

**DOI:** 10.7759/cureus.32752

**Published:** 2022-12-20

**Authors:** Dorothy Ibifuro Makanjuola, Shrouq Solimanie, Abdulmohsen Al Kushi, Najd Al Luhaydan, Reena Alharbi

**Affiliations:** 1 Medical Imaging, King Abdulaziz Medical City Riyadh, Riyadh, SAU; 2 Medical Imaging, Ministry of National Guard for Health Affairs - Central Region, Riyadh, SAU; 3 Pathology and Laboratory Medicine, Ministry of National Guard for Health Affairs - Central Region, Riyadh, SAU

**Keywords:** gynecomastia, male breast cancer, female pattern, breast cancer, mammography

## Abstract

Introduction

Male breast cancer is rare, accounting for about 1% of total breast cancer cases. In contrast to gynecomastia, which has a painful, soft, movable mass concentric to the nipple, the traditional presentation is a painless, hard, eccentric retro-areolar mass. However, when cancer occurs concurrently with significant gynecomastia, the mammographic pattern simulates female-type breast cancer, whereby there is a variable location and pattern of cancer. This study addresses the clinical and radiologic implications of this combination of gynecomastia and co-existing breast cancer. This combined presentation has not been highlighted thus far.

Materials and method

Following institutional approval, a retrospective study of male breast cancer was conducted over a 10-year period (2011-2021) in a single institution. Age, clinical presentation, risk factors, comorbidities, imaging results, and comprehensive pathology reports were all obtained from the picture archiving and communication system (PACS). Patients who did not have an initial imaging examination were eliminated from the study.

Results

There were 17 cases in all that were investigated. Nine of the men exhibited a classic presentation appearance, whereas eight had gynecomastia. The mean age was 58 years. The female-type presentation included multicentric cancers away from the nipple, diffuse parenchymal involvement, leukemia/lymphoma, and positive axillary lymphadenopathy without intramammary lesion, some of which had delayed investigation due to clinical suspicion of gynecomastia or breast swelling. All of the radiologic diagnoses were accurate. The pathology report in all except two cases was hormone receptor-positive and human epidermal growth factor receptor 2 (HER2) negative.

Conclusion

Female-type presentation of male breast cancer is highlighted to prevent false clinical impressions and delayed radiologic investigation and treatment. Mammography readily identifies such cancers and should be requested at the initial clinical presentation of males with significant gynecomastia or risk factor.

## Introduction

Male breast cancer is rare, representing less than 1% of all breast cancer [1-3]. It is therefore less widely known and understood than female breast cancer. The mean age at diagnosis of male breast cancer is said to be 67 years which is 5-10 years older than the female average at diagnosis of female breast cancer in the Western population. Also, the worldwide variation simulates that of female breast cancer which has a higher rate in the Western population as compared with those of Asia [4-6]. Similar to female breast cancer, the majority of patients with male breast cancer had no risk factors. When present, the risk factors in most are the same as female breast cancer, e.g., advanced age, obesity, chest radiation, apart from gender-related factors. Klinefelter syndrome with 47,XXY chromosome is the strongest risk factor and is 20-50 times higher than the normal 40,XY. The risk is also relatively higher than in men with first-degree relatives and those with breast cancer gene mutation 2 (BRCA2) rather than breast cancer gene mutation 1 (BRCA1) [7-10].

The classic presentation in the text is a painless retroareolar eccentric mass in contrast to gynecomastia which has a soft painful, mobile retroareolar mass concentric to the nipple. Microcalcification is rare in male breast cancer and, when it occurs is coarse and less linear. Supportive features of male breast cancer are nipple discharge, retraction and skin changes [11-13].

This classic location of presentation is determined by the origin of the tumor from the epithelial components of the primitive ducts following the involution of the male breast tissue at birth [3]. However, when male breast cancer occurs concomitantly with significant gynecomastia, the location of cancer and its pattern could simulate the various patterns of female breast cancer.

The coexistence of gynecomastia with male breast cancer is well known even at autopsy, and some indicated that it did not compromise cancer detection at mammography [14-17] However, a clear description of this pattern and its potential implications have not been emphasized. possibly because male breast cancer is rare. This communication, therefore, wishes to highlight the female-type presentation of male breast cancer, identify the clinical and radiologic implications and advice accordingly.

## Materials and methods

A retrospective study of pathologically diagnosed male breast cancer in a single institution over a period of 10 years (2011-2021) was made. Approval from the institution’s research committee was obtained (study number RC18.121.R). Inclusion criteria were cases of male breast cancer with clinical, radiologic, and pathologic data obtained from the hospital picture archiving and communication system (PACS). Cases diagnosed elsewhere and receiving treatment in the institution without initial imaging pictures were excluded. The standard departmental protocol for imaging the male breast was applied. This involved mammography as the first-line procedure in males older than 26 years, followed by complementary breast ultrasound. MRI is employed when mammography and ultrasound are indeterminate or when it is considered necessary to search for occult breast cancer, as occurred in one of the cases. PETCT scan is applied for the detection and evaluation of metastatic cancer as well as in cases of lymphoma or leukemia. Age distribution was analyzed (Table 1).

**Table 1:** Age distribution. The total number analyzed was 17, with the 17-year-old patient with leukemia excluded. The mean age of the 17 patients was 59 years.

Age range	Number (N=18)
40-50	3
51-60	4
61-70	5
71-80	5

**Figure 1:**
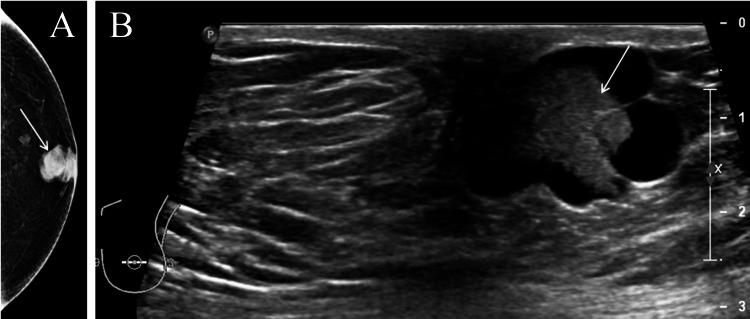
A 66-year-old male with nine months history of a breast lump (arrowed). Mammograms reveal classic a retroareolar eccentric mass shown in ultrasound as an intracystic lesion (arrowed). Pathology confirmed papillary cancer. Hormone receptor-positive and human epidermal growth factor receptor 2 (HER2) negative. (A) Mammogram - craniocaudal view. (B) Ultrasound of retroareolar region.

**Figure 2:**
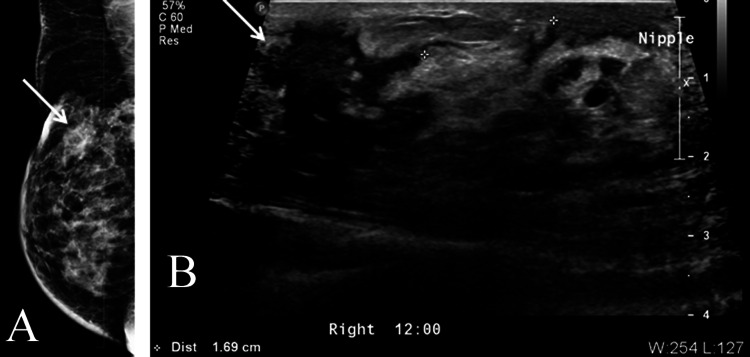
A 55-year-old male with right breast mediolateral oblique mammograms showing prominent gynecomastia concomitant with very suspicious mass (breast imaging-reporting and data system [BI-RADS] 5) at the upper outer quadrant (arrowed) with skin retraction confirmed in ultrasound, invasive ductal carcinoma, hormone receptor-positive and human epidermal growth factor receptor 2 (HER2) negative. (A) Right breast mammogram mediolateral oblique view. (B) Corresponding ultrasound of the right breast.

Two groups of patients were identified. Those with a classic presentation of breast cancer without associated gynecomastia, (i.e., single mass, largely central in location) (Figures 1A, 1B) and those with associated gynecomastia (Figures 2A, 2B).

 

The data related to the two groups were obtained and compared with each other (N=18) (Table 2).

**Table 2:** Comparative analysis of the findings in the classic pattern and female-type presentation of male breast cancer.

Pattern of mammography presentation	Earlier presentation without x-ray	Duration of symptoms	Presence of metastasis on presentation, including axillary lymph node	Initial clinical impression	Correct diagnosis
Classic pattern total number N = 9 (Figure 1)	Nil	Between 6-9 months	2	Breast lump/mass = 8, breast swelling = 1	8 out of 9
Female-type presentation male breast cancer N = 8 (Figure 2)	4 including 1 assessed as inflammation	18 months to 3 years	6	Breast mass = 1, breast swelling = 3, breast inflammation = 1, axillary lymph with negative breast lesion = 1	3 out of 8

**Figure 3:**
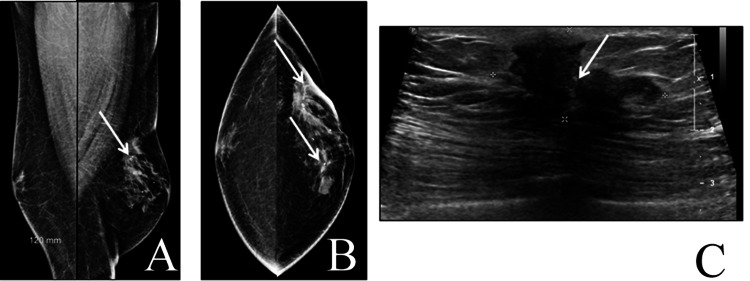
A 60-year-old male with left breast swelling of three years' duration. Mammograms revealed multicentric spiculate masses at the left upper outer quadrant with skin retraction and lower inner quadrant (arrowed) considered BI-RADS 5 and also confirmed in ultrasound. Staging revealed axillary, lung, and bone metastases. Biopsy as well as modified radical mastectomy confirmed multicentric lesions, invasive ductal carcinoma, hormone receptor-positive and human epidermal growth factor receptor 2 (HER2) negative. (A) Bilateral mediolateral oblique mammograms. (B) Bilateral craniocaudal mammograms. (C) Corresponding ultrasound of the left upper quadrant.

**Figure 4:**
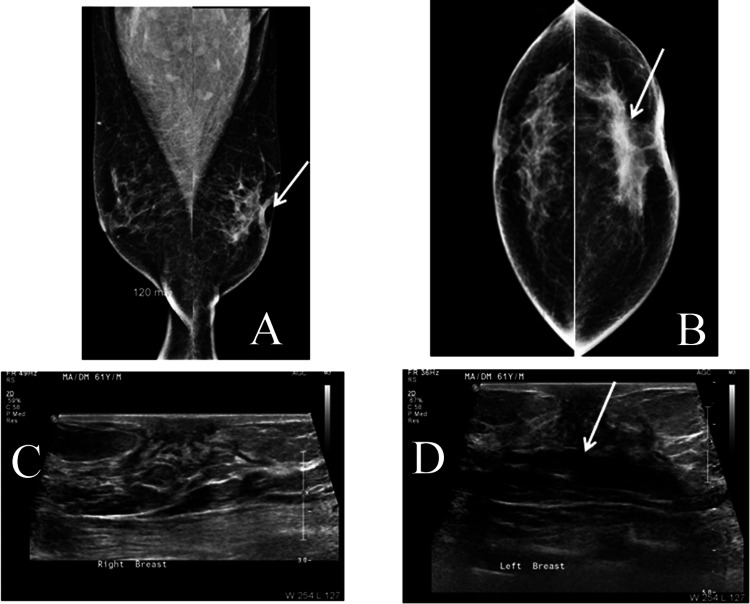
A 62-year-old male with three years history of left breast swelling and a few months of periareolar thickening. Breast inflammation was clinically suspected. Mammograms revealed left diffuse parenchyma changes with generalized asymmetric density. No masses were seen even in ultrasound. The pattern simulates early inflammatory breast cancer. Biopsy and radical mastectomy revealed invasive ductal carcinoma, hormone receptor-positive and human epidermal growth factor receptor 2 (HER2) negative. (A) Bilateral mammograms in mediolateral oblique view. (B) Bilateral mammograms in craniocaudal view. (C) Ultrasound of the right breast. (D) Ultrasound of the left breast.

The presence of risk factors, including a family history of breast cancer, and gene mutation of breast cancer 1 and breast cancer 2, were also recorded. The full histopathology of the patient was studied. Four-view mammograms were obtained in the usual manner with a Hologic tomosynthesis machine and the findings were classified according to the BI-RADS classification of lesions [18]. The presence or absence of gynecomastia along with the suspicious lesions was recorded. The female-type presentation was considered when there was concomitant gynecomastia. The lesions identified had variable locations including multicentricity (Figures 3A-3C). The suspicious lesions were described as masses including the pattern of the outline, e.g., speculation or angular. Others were parenchymal distortion, focal asymmetry, diffuse breast changes (Figures 4A-4D), and microcalcification with or without associated masses.

The classic pattern was considered as those with a single mass, mostly central in a location with various outlines. Ultrasound was performed with Philips equipment (Epiq 7G) and the findings were described in the usual breast imaging-reporting and data system (BI-RADS) [18] pattern of classification.

Emphasis was placed on mammography because it is the most accurate way of assessing the male breast and indeed the most widely available imaging modality for the breast. Staging examinations were obtained with chest and abdominal computed tomography (CT) and bone scan. Positron emission tomography (PET) scan and magnetic resonance imaging (MRI) was performed on occasions namely cases with positive axillary lymph nodes with no breast lesions.

## Results

**Figure 5:**
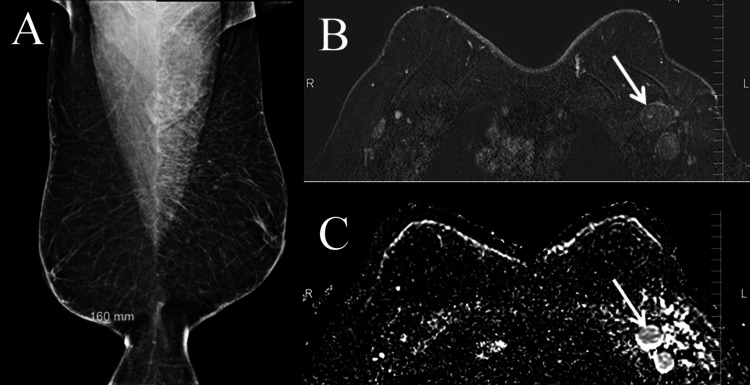
A 48-year-old male who presented with axillary swelling for nine months. No significant findings in blood analysis. Chest and abdominal CT only revealed suspicious left axillary lymph nodes. Biopsy of the lymph nodes identified metastatic invasive ductal carcinoma. Subsequent mammograms, ultrasound, breast MRI, and PET CT revealed large fatty gynecomastia with no breast lesions except positive axillary lymph nodes. (A) Bilateral mammograms in mediolateral oblique view. (B) Breast MRI contrast-enhanced subtraction view. (C) PET CT.

**Figure 6:**
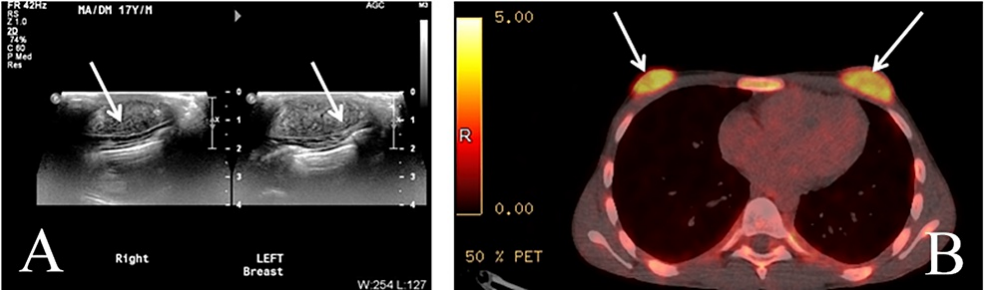
A 17-year-old male in remission from lymphoblastic leukemia when he developed bilateral breast swelling. The clinical suspicion was gynecomastia. Bilateral breast ultrasound revealed bilateral symmetrical well-defined heterogeneous retroareolar concentric masses also revealed in PET CT as bilateral symmetric retroareolar masses. Due to the clinical setting of leukemia, leukemic infiltrates were suspected and coded as BI-RADS 4C. A bilateral biopsy revealed lymphoblastic leukemia. (A) Bilateral retroareolar ultrasound. (B) PET CT.

Of the 18 cases of breast cancer detected, nine had the classic presentation, while eight cases, including one with metastatic axillary lymphadenopathy with no identified breast lesion (Figures 5A-5C), did not have the classic presentation. There was also one case of bilateral retro areolar leukemia (Figures 6A, 6B), which was excluded from the analysis.

The clinical impression of those with the classic mammographic presentation of solitary central mass was correct in almost all, with the correct diagnosis of breast cancer in eight out of nine cases, while the clinical interpretation of those with associated gynecomastia was that of benign findings such as gynecomastia, breast swelling, and breast inflammation with the correct diagnosis of three out of eight cases.

Risk factors

Two cases had BRCA2 positivity. One showed a variant of uncertain significance. One patient had a positive family history of a 43-year-old sister with breast cancer with no known gene mutation. Notably, the two positive cases of BRCA2 were relatively young (41 and 54 years old), and one of them had prostatic cancer.

Pathology reports

Fifteen cases had intraductal carcinoma (three with associated ductal carcinoma in situ). There was one case of papillary carcinoma and one of primary breast lymphoma. There was also a case of leukemia, which was excluded from statistical analysis. No case of lobular carcinoma was found. All the cancer cases except one were hormone receptor-positive and HER-2 negative. The incidence of metastasis was higher in the group with the female-type presentation of male breast cancer, likely due to the delay in diagnosis.

## Discussion

The study revealed that about 47% of the patients studied with male breast cancer had concomitant gynecomastia and six of the 17 had significant gynecomastia to simulate the female-type presentation of male breast cancer. This incidence of concomitant gynecomastia is consistent with the reports in the literature [16,17,19] although the mammographic pattern is not described. The combined occurrence is thought to be due to elevated estrogen levels which are seen in both conditions, although the advanced presentation of male breast cancer is well recognized.

Advanced presentation of male breast cancer can be related to lack of screening which is not recommended in males and the lack of awareness about the occurrence of breast cancer in males. However, since male breast cancer normally presents in a clinical setting, there is reliance on correct clinical assessment and some clinicians would not request mammograms unless malignancy is suspected.

In a setting of significant gynecomastia, cancers located away from the usual retro-areolar location could be misinterpreted as breast swelling as occurred in Figures 2A, 2B. In the rare case of fatty breast or pseudo gynecomastia who presented with metastatic axillary lymphadenopathy with no obvious breast lesion (Figures 5A-5C). Breast cancer was totally unsuspected until the axillary node biopsy revealed metastatic breast cancer. His MRI evaluation was negative for a breast lesion. No other lesions were detected at CT or PET CT. Also, in the younger 17-year-old male with bilateral breast leukemia (Figures 6A, 6B), the clinical impression was gynecomastia.

Metastatic axillary lymphadenopathy without identified breast cancer is an uncommon occurrence even in females. It is interesting that we found one case in a male patient (Figures 5A-5C). The mean age of the patients was 59 years, with a range of 43 to 82 years, excluding the patient with leukemia.

The majority of the subjects studied did not have any of the established risk factors for breast cancer which is consistent with the literature in both males and females. Only three patients had the gene mutation, two with BRCA2 and one with a variant of uncertain symptoms. The patients with BRCA2 were relatively young. This involvement of relatively young patients is consistent with earlier studies of male patients with breast cancer [7,20,21].

The pathologic reports revealed a rather uniform pattern. All but two had invasive ductal carcinoma, with a single case of papillary cancer and one case of primary breast lymphoma. A case of leukemia in a 17-year-old was also described, due to the unusual pattern of presentation with bilateral symmetrical retro-areolar mass. Papillary cancer is said to be the second type of breast cancer in males, and leukemia is known to be metastatic to the male breast although the most common is from the prostate. All but one of the patients with invasive ductal carcinoma as well as the patient with papillary cancer were hormone receptor-positive and HER2-negative.

Limitations of the study

This investigation suffers from the limitation of being a retrospective study. For example, the report of some of the genetic studies was not found in some of the cases. Also, some of the cases presented without any initial imaging and some died of metastatic disease, which makes the prevalence rate difficult to determine.

## Conclusions

Female-type presentation of male breast cancer is an uncommon and significant radiologic presentation of male breast cancer. It is a potential cause of false clinical impressions with consequent delay in diagnosis and treatment. Mammography is suggested for male patients with breast mass, prominent gynecomastia, or clinical breast swelling to reveal any concomitant cancer.
